# Non-invasive brain stimulation in cognitive sciences and Alzheimer's disease

**DOI:** 10.3389/fnhum.2024.1500502

**Published:** 2025-01-14

**Authors:** Claudia Carrarini, Chiara Pappalettera, Domenica Le Pera, Paolo Maria Rossini

**Affiliations:** ^1^Department of Neuroscience and Neurorehabilitation, IRCCS San Raffaele Roma, Rome, Italy; ^2^Department of Theoretical and Applied Sciences, eCampus University, Como, Italy

**Keywords:** non-invasive brain stimulation, dementia, connectivity, neurorehabilitation, cognitive sciences

## Abstract

Over the last four decades, non-invasive brain stimulation techniques (NIBS) have significantly gained interest in the fields of cognitive sciences and dementia care, including neurorehabilitation, for its emerging potential in increasing the insights over brain functions and in boosting residual cognitive functions. In the present paper, basic physiological and technical mechanisms and different applications of NIBS were reviewed and discussed to highlight the importance of NIBS in multidisciplinary and translational approaches in clinical and research settings of cognitive sciences and neurodegenerative diseases, especially in Alzheimer's disease. Indeed, NIBS strategies may represent a promising opportunity to increase the potential of neuromodulation as efficacious interventions for individualized patients care.

## Introduction

Non-invasive brain stimulation (NIBS) boasts a relatively recent history, reaching the attention of scientific and clinical communities with the advent of electroconvulsive therapy in the 1940s for treating several mental disorders (Kellner et al., 2012). Apart from electroconvulsive therapy, following pioneering studies in primates and human volunteers (Gualtierotti and Paterson, 1954) early abandoned for serious side-effects, non-invasive high-voltage electrical brain stimulation finally emerged in the early 80s (Merton and Morton, 1980). This new technique, in its different modalities (Merton and Morton, 1980; Rossini et al., 1985a,b, 1987a; Day et al., 1987), remained mainly confined to research laboratories with limited clinical uses in diagnosis and intraoperative monitoring (Day et al., 1987; Rossini et al., 1988) due to possible side-effects and painful nature of the procedures. A completely new impetus was injected in this field with the discovery of transcranial magnetic stimulation (TMS) by Barker et al. (1985). In this technique, when a coil carrying high-intensity electrical stimulation is placed on the scalp, it generates a transient and strong magnetic field around the coil's circumference which indirectly induces an electric current under the skull, flowing in the opposite direction to that circulating within the coil. In more detail, when the primary motor cortex is targeted by TMS, the resulting activation of the corticospinal tract, if the stimulus intensity overcomes the threshold for spinal alpha-motoneuronal depolarization, elicits a motor response in the target muscle (usually forearm/hand and leg/foot) known as motor evoked potential (MEP). This method is primarily used to assess cortical excitability and motor corticospinal pathways viability (i.e., the Central Motor Conduction Time, CMCT). The process is painless and associated with minimal or no adverse effects.

In addition, magnetic seizure therapy by repetitive TMS (rTMS) was therefore introduced for the recovery from psychiatric disturbances, such as resistant major depressive disorders (Lisanby et al., 2001).

**Figure 1:**
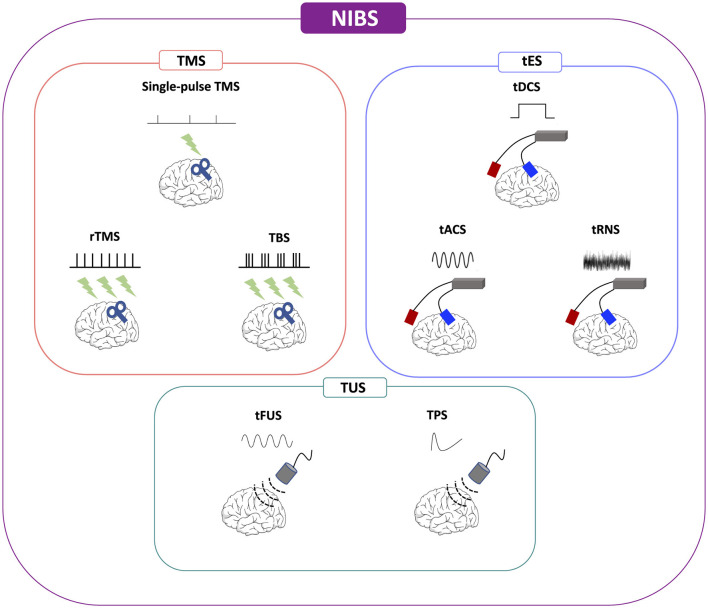
Non-invasive brain stimulation (NIBS) methodologies. NIBS, non-invasive brain stimulation; tACS, alternating current stimulation; tDCS, transcranial direct current stimulation; tFUS, transcranial focused ultrasound stimulation; tRNS, random noise stimulation; rTMS, repetitive transcranial magnetic stimulation; TMS, transcranial magnetic stimulation; TPS, transcranial pulse stimulation; TBS, theta-burst stimulation; TUS, transcranial ultrasound stimulation.

**Figure 2:**
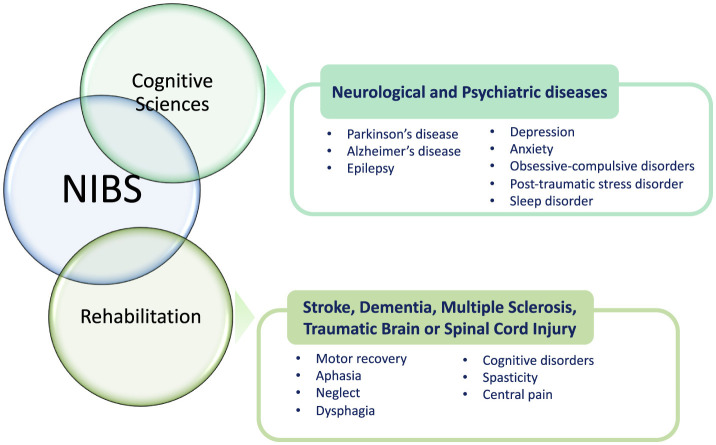
Fields of applications for non-invasive brain stimulation (NIBS) in neuroscience.

Over the last four decades, NIBS techniques have continuously included different methodologies and gained attention in basic and translational research and clinical settings (both for diagnosis and treatment/rehabilitation) due to their favorable profile of efficacy and safety (Figure 1). Besides providing important functional information on the role of the stimulated brain area in research protocols, these procedures have shown promising results also in treating neurological (e.g., dystonias, dyskinesias, cognitive deficits, chronic pain) and psychiatric disturbances (e.g., drug-resistant major depression; Koch, 2013; Ren et al., 2014; Cho and Hallett, 2016; Gunduz et al., 2017) as well as in exploring brain functions including cognition, perception, and motor control (Bonnì et al., 2015; Sprugnoli et al., 2017). Perturbation-based and neuromodulatory interventions by NIBS approaches have been applied to determine a transient modification in regional brain dynamics related to insight processes, also to enhance insight problem-solving skills (Sprugnoli et al., 2017). Therefore, during the last years, different NIBS techniques have been developed and explored also in individuals suffering from cognitive disturbances and dementia. In general, NIBS serves as a versatile tool across both cognitive sciences and rehabilitation, offering promising applications in various neurological and psychiatric conditions (Figure 2). In cognitive sciences, NIBS has gained attention for its potential to explore and treat disorders such as Parkinson's disease, Alzheimer's disease (AD), and epilepsy, as well as mental health conditions including depression, anxiety, obsessive-compulsive disorder, post-traumatic stress disorder, and sleep disorders. This approach allows researchers and clinicians to investigate cognitive functions and address the symptoms associated with these conditions by modulating brain activity in targeted regions. In the field of rehabilitation, NIBS is used to support recovery from neurological impairments resulting from stroke, dementia, multiple sclerosis, traumatic brain injury, and spinal cord injury. By stimulating specific brain networks, NIBS can facilitate motor recovery, language function, and attention, helping to address challenges such as aphasia, spatial neglect, and dysphagia. Additionally, it shows promise for managing cognitive disorders, spasticity, and chronic pain, which are common in these conditions. Through its dual role in advancing cognitive research and enhancing rehabilitative outcomes, NIBS represents a powerful approach for both understanding and alleviating the effects of neurological and psychiatric disorders.

Moving toward cognitive disorders, AD represents the most common cause of dementia worldwide and its management has been defined as a global public health emergency by the World Health Organization (WHO) and the Alzheimer's Disease International (ADI; Lane et al., 2018; Knopman et al., 2021). Nowadays, available therapeutic strategies for AD and other dementias may only provide minimal symptomatic relieves, without arresting the underlying pathological disease course. In this frame, non-pharmacological interventions including lifestyle modifications (e.g., physical activity, healthy diet, cognitive training, etc.) and NIBS have been increasingly explored as potential candidates for mitigating cognitive dysfunctions and potentiating shaping/reinforcing neural networks for resilience to progression in individuals with cognitive decline (Imtiaz et al., 2014; Gonsalvez et al., 2017; Livingston et al., 2024). Considering the complex and heterogenous substrate of dementia, involving a cascade of causative factors (e.g., brain protein aggregations, neuroinflammation, synaptic transmission failures, oxidative stress; Licher et al., 2019; Peters et al., 2019; Rossini et al., 2020), it is unlikely that a single therapeutic/rehabilitative approach may be sufficient and satisfactory to prevent or treat cognitive decline. Therefore, NIBS techniques have increasingly been applied as single or add-on therapies (i.e., combined with pharmacological drugs) for people suffering from dementia for their properties of easily and non-invasively modulate neuronal activity and influence cognitive processes (Teselink et al., 2021).

In this review of literature, fundamental concepts on NIBS and their main and general results in the fields of cognition and AD are discussed to emphasize the promising standing of these techniques in clinical and research protocols.

## Basic mechanisms of NIBS

Even though NIBS refers to a wide range of devices aimed at modulating neuronal activities and networks, TMS and low-intensity transcranial electrical stimulation (tES) are the most widely used techniques (Figure 1).

As aforementioned, TMS delivers via a coil a transient magnetic field to the scalp, which creates in the brain a brief electric impulse leading to either trans-synaptic depolarization or hyperpolarization of cortical neurons via cortical interneuronal excitation. The effects of TMS are influenced by different technical parameters, such as coil type or angle to the scalp, intensity, focality, and frequency/pattern of stimulations, as well as subject-specific factors like age/height and the presence of pharmacological agents including drugs (Rossini et al., 2010; Miniussi and Rossini, 2011; Hartwigsen and Silvanto, 2023). rTMS, introduced in the late 1990s, delivers rhythmic trains of impulses, in rapid succession at high frequency [up to 100-Hertz (Hz) repetition rate; Ren et al., 2014]. Typically, low frequency rTMS ( ≤ 1 Hz) is considered to inhibit targeted brain regions and their associated networks, whereas high frequency rTMS (>5 Hz) is used to increase cortical excitability (Ren et al., 2014). Interestingly, rTMS is more effective at modulating neuronal activity than single-pulse TMS, likely due to synaptic recruitment and shaping/stabilization of new neural networks if repeated across successive sessions and days (Miniussi and Rossini, 2011). Recent research highlights the efficacy of rhythmic TMS protocols, which have been shown to positively influence both cognitive and motor performance. In their pivotal study, Hanslmayr et al. revealed that the phase of a 7 Hz oscillation prior to the onset of visual stimuli significantly predicted both perceptual performance and the effective connectivity between higher-level and lower-level visual processing regions. Specifically, they demonstrated that the phase of the 7 Hz oscillation influenced bidirectional information flow between the left lateral occipital cortex and the right intraparietal sulcus, utilizing advanced techniques like psychophysiological interaction and dynamic causal modeling (Hanslmayr et al., 2013). For example, brief bursts of high-rate repetitive stimulation at 5 Hz (the theta-burst of EEG rhythm) represent the most popular type of “patterned” TMS which has a strong ability to drive synaptic plasticity. Theta-burst stimulation (TBS) is a newer form of patterned rTMS which can be delivered at three very short bursts with high frequency of typically ~50 Hz, repeated at 5 Hz intervals. Compared to rTMS, it is characterized by shorter stimulation time and lower intensity. TBS may be applied as intermittent (iTBS) or continuous (cTBS) pulses, which show opposite effects: in cTBS, pulses are delivered without any interruption, inducing long-term depression (LTD)-like effects, whereas in iTBS trains are repeated every 10 s for 190 s, leading to long-term potentiation (LTP)-like effects (Chung et al., 2015, 2016; Suppa et al., 2016).

Additionally, cortico-cortical paired associative stimulation (ccPAS) represents another innovative neurostimulation technique that leverages the principles of Hebbian plasticity to modulate connectivity between cortical areas. ccPAS involves paired TMS pulses delivered to different cortical sites at specific interstimulus intervals. In their recent review, Tarasi et al. summarized the growing body of ccPAS research focused on visual perception. Their findings illustrated that ccPAS enhances functional connectivity within the visual system, significantly impacting motion perception, emotion recognition, and metacognitive judgments (Tarasi et al., 2024). Additionally, Rizzo et al. demonstrated that ccPAS can effectively influence interhemispheric inhibition and improve fine motor control in healthy individuals (Rizzo et al., 2011). This suggests its applicability in developing rehabilitation strategies aimed at restoring motor function following neurological events. Studies have demonstrated that ccPAS can influence functional connectivity in various networks, impacting cognitive and motor functions. For example, Fiori et al. provided further evidence that ccPAS enhances the pathway from the ventral premotor cortex to the primary motor cortex, which is critical for skilled object-oriented hand actions (Fiori et al., 2018). Their findings imply that targeted stimulation could lead to improved performance in tasks requiring fine manipulation, highlighting the relevance of ccPAS in enhancing motor skills. Di Luzio et al. explored how ccPAS shapes perceptual sensitivity and metacognitive skills, revealing distinct neural networks that underlie these functions. This work has significant implications for cognitive training and therapeutic interventions in conditions that affect perceptual and cognitive processing (Di Luzio et al., 2022). Furthermore, Di Lorenzo et al. investigated ccPAS in AD, uncovering impairments in long-term potentiation-like plasticity mechanisms. This emphasizes the potential of ccPAS to facilitate plasticity and recovery, particularly in populations with cognitive impairments (Di Lorenzo et al., 2018).

tES releases an electrical current of extremely low intensity [in the order of few milliAmperes (mA)] through the brain by two or more large surface electrodes applied to the scalp. Its neuromodulatory effects rely on the membrane polarization defined by the position of an excitatory anode and an inhibitory cathode. Differently from TMS, tES is not powerful enough to elicit an action potential (i.e., MEP). This technique encompasses different methods such as transcranial direct current stimulation (tDCS), alternating current stimulation (tACS), and random noise stimulation (tRNS; Paulus et al., 2016). tDCS delivers a one-way direct electrical flow at low intensities (between 0.5 and 2 mA) while tACS and tRNS use an alternating electrical current, the first with a specific stimulation frequency, while the latter with random frequencies (Antal et al., 2022). More recently, following the original methods developed by Rossini et al. with “unifocal transcranial electrical stimulation” (Rossini et al., 1985a, 1987b), high-density multi-electrode tDCS arrangements have been introduced to be used to simultaneously stimulate multiple brain regions. Of note, an algorithm for determining the optimal placement and current output of multifocal tDCS electrodes has also been designed (Fischer et al., 2017). A novel approach for multichannel, network-targeted tDCS (net-tDCS) have been tested using a Magnetic Resonance Imaging (MRI)-compatible multichannel tES device, suggesting the possibility of controlling network connectivity patterns to enhance cognitive processes and therapeutic effects (Mencarelli et al., 2020).

Lately, a novel technique based on alternating electric current stimulation at kilohertz frequency field stimulation (kHz-FS) has been introduced considering its properties in targeting deep brain structures and in modulating neural activity without any main side effect. The kHz-FS (>1 kHz) can be used for transcranial brain stimulation, spinal cord stimulation, deep brain stimulation, and peripheral nerve stimulation. The majority of studies have been conducted on animal models or *in vitro*, with still limited evidence in humans and no safety limits for clinical application have been defined. Different neuronal responses have been observed with kHz-FS, categorized into subthreshold, suprathreshold, synaptic and thermal effects, with various mechanisms of action (i.e., facilitation, desynchronization, spike-rate adaptation, conduction block, non-monotonic activation, and synaptic fatigue; Neudorfer et al., 2021).

Regarding TMS, MEP parameters are considered effective measurements for objectively assessing brain excitability (e.g., resting and active Motor Threshold; Rossini et al., 1994, 2015). They are also valuable for measuring corticospinal tract functionality, owing to the high reproducibility of MEP under standardized conditions, for example in order to measure the CMCT of impulse propagation from the motor cortex to the spinal myelomers governing the target muscle (Rossini et al., 1994, 2015).

The recent development of innovative types of NIBS, namely transcranial focused ultrasound stimulation (tFUS) and transcranial pulse stimulation (TPS), carries novel interest as neuromodulation methods (see Figure 1). Respect to other NIBS techniques, transcranial ultrasound stimulation (TUS) methods are characterized by programmable depth of stimulation focus on brain areas and more accurate millimetric spatial resolution (Lee et al., 2024). While high-intensity tFUS produces a caloric effect at the focus level with a lesional effect on the neurons at the focus center (this is proposed as a non-invasive alternative to Deep Brain Stimulation methods), low-intensity tFUS has successfully been applied to humans to modulate neuronal excitability with millimetric precision also in deep cerebral structures and to produce effects beyond the period of stimulation in motor, visual and cognitive functions despite recent challenges due to the variability of such effects (Walsh and Cowey, 2000; Huang et al., 2022). Low-intensity tFUS uses kHz frequencies and, penetrating biological tissues (i.e., skull, brain), can safely stimulate or inhibit targeted neuronal activity. The main mechanism of action is based on micro-mechanical pressure waves induced by pulsed ultrasound on ion channels (Kubanek, 2018). TPS releases repetitive, single, and ultrashort mechano-acoustic pulses, namely shock waves, of low frequency (4 Hz) and short duration (3 μs) that are repeated every 200–300 ms. Shock waves promote mechanotransduction, stimulating the release of vascular endothelial growth factors (VEGF) and nitric oxide (NO) improving blood flow and inducing neoangiogenesis. Its energy is discharged with high spatial precision through a MRI-driven neuro-navigated device to identify pre-defined brain areas with real-time monitoring capabilities (Beisteiner et al., 2020). This method offers an advantage over tFUS by mitigating tissue warming and standing waves through the utilization of very short pulses devoid of periodic waves or prolonged sonication trains. Standing waves have the potential to induce unintended secondary stimulation maxima, thereby constraining the spatial specificity of tFUS. Furthermore, TPS stands out as the inaugural ultrasound-based NIBS technique approved for clinical applications.

The use of NIBS has gained general acceptance with the establishment of practical, safety and ethical guidelines over the years. As NIBS is used increasingly on both healthy subjects and patients, safety and risk management continue to be critically updated (Rossi et al., 2009, 2021; Groppa et al., 2012; Lefaucheur et al., 2014; Rossini et al., 2015; Antal et al., 2017; Fried et al., 2021). Currently, the most significant adverse effect associated with NIBS (especially TMS) is the occurrence of seizures, which are extremely rare and primarily occur in epileptic subjects or those taking medications that lower the seizure threshold and during repetitive or patterned stimulation types (i.e., rTMS, TBS). Despite the low risk level, rigorous patient monitoring and safety precautions, and a high personnel expertise and adherence to ethical standards are recommended (Fried et al., 2021; Rossi et al., 2021).

## NIBS in cognitive sciences

Non-invasive brain stimulation techniques have extensively been explored as therapeutic tools for a variety of medical conditions, including relieving depression or enhancing cognitive functions in neurodegenerative diseases (Figure 2). Research has also employed these methods to probe the neural bases of cognitive processes and to develop therapeutic strategies for neurological and psychiatric conditions. Therefore, NIBS techniques have largely been used in the field of cognitive neurosciences due to their ability to selectively interfere with one specific brain area involved in a task, thus allowing the examination of its role in that cognitive performance.

The application of NIBS in cognitive research began with the pioneering study by Amassian et al., which demonstrated that single-pulse TMS could disrupt visual perception with high temporal precision (Amassian et al., 1989). This work established TMS as a tool for creating “virtual” brain lesions, enabling researchers to investigate the causal relationships between specific brain regions and cognitive functions. Over the past two decades, the use of NIBS has evolved from this point, based on directly linking cognitive processes to brain regions, to the understanding of how NIBS interacts with brain activity. The interaction between NIBS and cognitive processes primarily depends on the effects induced on cortical and subcortical networks connected to the stimulated area, rather than on the excitation or inhibition of a specific brain region (Miniussi and Rossini, 2011).

Therefore, TMS has been employed to temporarily halt speech production, or interrupt the occurrence of voluntary movement (Amassian et al., 1989; Day et al., 1989; Pascual-Leone et al., 1991) or modulate encoding/retrieval memory processes (Rossi et al., 2001). Moreover, rTMS can contribute to cognitive studies in two distinct modalities: “on-line,” when it is applied during a cognitive task, and “off-line,” when it is used immediately prior/after cognitive performance.

In general, NIBS methods can be applied to large cohorts of subjects and results can be compared with control groups, including placebo/sham conditions. The possible use of neuronavigation allows for direct interaction with targeted brain areas involved in a cognitive task and enables stimulation at different time points to precisely study the flow and inner time hierarchy of a network-related cognitive process. In general, a neuronavigation system allows for targeting cortical areas in a more accurate and reproducible way of stimulation. Neuronavigation couples NIBS methods with the specific MRI scan of the patient head allowing a real-time tracking of the TMS coil on the patient brain with millimetric precision. To associate the MRI scan with the subject's head, defined landmarks on the MRI images are manually selected on the patient head by using a digitizing pen. Then, the coil (provided with optical trackers) and the site of stimulation can be easily visualized over the brain MRI model (Lioumis and Rosanova, 2022).

Cognition is subserved by several neuronal circuits, which can be more or less receptive to NIBS techniques. For instance, deeper structures (e.g., hippocampus, cingulate gyrus) may be more insensitive to such techniques, whereas the dorsolateral prefrontal cortex (DLPFC), involved in working memory, may benefit from these stimulations (Rossi et al., 2004; Brunoni and Vanderhasselt, 2014). Hence, NIBS techniques have extensively been applied to explore higher brain functions, such as memory, executive function, attention, and language (Sprugnoli et al., 2017; Saccenti et al., 2024), with heterogenous results. Before analyzing NIBS applications in the field of AD, the main findings of cognitive sciences in healthy control (HC) subjects are presented in this section (Table 1) to explore the general effects of these methodologies on cognition.

**Table 1:** Non-invasive brain stimulation studies in cognitive sciences and healthy individuals.

**References**	**Sample**	**NIBS technique**	**Target site**	**Main results**
**Transcranial Magnetic Stimulation**				
Amassian et al. (1989)	4 HC	TMS	Occipital cortex	Disrupting visual perception
Bonnì et al. (2015)	30 HC	cTBS	Precuneus, posterior parietal cortex, and vertex	In respect to the stimulation of posterior parietal cortex and vertex, cTBS on the precuneus induced a selective decrease in source memory errors, indicating an improvement in context retrieval
Day et al. (1989)	8 HC	TMS	Vertex	Delay in the execution of the movement
Koch et al. (2005)	9 HC	rTMS	Posterior parietal cortex, premotor cortex and DLPFC of the right hemisphere	Two different networks coexist in DLPFC, as follows: a local network subserving the decisional processes and a second network functionally interconnected with the posterior parietal cortex and activated when a certain spatial information has to be memorized
Oliveri et al. (2004)	8 HC	TMS	Primary motor cortex	Activation in the motor cortex increased for action words compared with non-action words but was not sensitive to the grammatical category of the word being produced
Oliveri et al. (1999)	17 HC	TMS	Frontoparietal region	Interhemispheric difference in the detection of cutaneous sensation, showing right hemispheric prevalence in the perception of contralateral as well as of ipsilateral stimuli
Rossi et al. (2001)	13 HC	rTMS	Primary motor cortex	rTMS transiently and safely interferes with the function of cortical networks involved in memory processes
Rossi et al. (2004)	66 HC	rTMS	Right and left DLPFC	In young subjects, rTMS of the right DLPFC interfered with retrieval more than left DLPFC stimulation. The asymmetry of the effect progressively vanished with aging, as indicated by bilateral interference effects on recognition performance
**Transcranial Electrical Stimulation**				
Cerruti and Schlaug (2009)	18 HC	Anodal/cathodal tDCS (sham-controlled study)	Left and right DLPFC	Anodal stimulation of the left DLPFC can improve complex verbal problem-solving performances
Chi and Snyder (2011)	60 HC	Anodal/cathodal tDCS (sham-controlled study)	Anterior temporal lobes	Only 20% of participants solved an insight problem with sham stimulation, whereas many participants solved insight problems with cathodal stimulation (decreased excitability) of the left anterior temporal lobe together with anodal stimulation (increased excitability) of the right anterior temporal lobe
Chi and Snyder (2012)	22 HC	Anodal/cathodal tDCS (sham-controlled study)	Anterior temporal lobes	None of the 22 participants in the main experiment solved the cognitive tasks before stimulation. But with 10 min of right lateralizing tDCS, more than 40% of participants could solved those tasks
Pergolizzi and Chua (2016)	58 HC	tDCS (sham-controlled study)	Posterior parietal and prefrontal cortex	Possible role of posterior parietal cortex in item and source memory retrieval, likely based on attentional and decision-making biases

A previous study with single- and paired-pulse TMS reported an activation of motor cortex when words associated with actions were recalled. This finding supports the hypothesis that lexical organization, encompassing all grammatical and semantic features, is represented across different and widespread brain areas. Therefore, retrieving a word with specific meaning may activate neural substrates linked to other circuits (e.g., sensori-motor area) beyond purely linguistic networks (Oliveri et al., 2004).

To compare the activity of the different brain areas in the delay and decision phases of a working memory task, trains of rTMS at 25 Hz were applied during the two phases (i.e., delay and decision phases) on different parieto-frontal areas in normal subjects. A pattern of TMS interference was found during the delay phase for both parietal and DLPFC sites of stimulation whereas, when rTMS was delivered during the decision phase, an interference was specifically shown for DLPFC. Therefore, the Authors postulated that two different networks coexist in DLPFC, as follows: a local neural network activated during decisional processes and a second network functionally interconnected with the posterior parietal cortex subserving the memory process (Koch et al., 2005).

NIBS techniques, especially tES, have widely been employed for associative memory, defined as the ability to remember the relationship between two unrelated items. The hippocampus and the surrounding medial temporal area are strictly involved in associative memory, although the storage and retrieval of memory representations are obtained through the interconnection of different neuronal networks, including also frontal and parietal lobes. Therefore, the stimulation of numerous cortical regions, in particular parietal cortex, may potentially induce network-wide effects on associative memory (Bjekić et al., 2023).

In the context of episodic memory, the role of prefrontal cortex (PFC) activation and lateralization during episodic memory tasks in physiological aging has been explored. rTMS was applied to the left or right DLPFC of two different groups of subjects (< 45 years and >50 years) engaged in visuospatial recognition memory tasks. The Authors observed an asymmetry of rTMS effects, which progressively diminished with aging. Specifically, in the younger group, the right DLPFC was more activated during memory recall compared to left DLPFC, whereas the predominance of the left DLPFC during encoding persisted in elderlies. This pattern is thought to reflect an age-related compensatory mechanism for episodic memory throughout normal aging (Rossi et al., 2004). Such hemispheric lateralization was investigated with TMS also for the perception of sensory stimuli with a right hemispheric prevalence in the perception of both ipsilateral and contralateral cutaneous stimuli, suggesting that right hemispheric damages could also affect the perception of ipsilateral sensory stimuli (Oliveri et al., 1999).

Also the posteromedial cortex, including the precuneus, has been supposed to be involved in episodic memory retrieval. cTBS was tested in HC individuals to support the role of the precuneus in the recognition memory. Interestingly, in respect to other stimulation sites, cTBS over the precuneus was associated to a selective reduction of source memory errors, demonstrating an improvement in episodic memory retrieval (Bonnì et al., 2015).

In another study, tDCS was bilaterally delivered over the posterior parietal cortex in comparison with prefrontal or sham stimulation during a recognitionc task followed by a source judgment. The parietal tDCS group showed a reduced false recognition, and less errors in item and source discrimination tasks in respect to those participants in the sham group. These findings supported the possible role of the posterior parietal cortex in item and source memory retrieval (Pergolizzi and Chua, 2016).

As regards problem-solving tasks, as compared to cathodal or sham stimulations, the anodal stimulation conducted with tDCS over the DLPFC increased the performances on a remote associates test (RAT), which represents a complex verbal task with associations to both creative thought and general intelligence. In an additional experimental subtest, the Authors verified the effect of an anodal tDCS on the right DLPFC, confirming the role of the left DLPFC stimulation for the improvement of complex verbal problem-solving performances (Cerruti and Schlaug, 2009). Similarly, other studies of tES over the temporal lobe reported increased cognitive performances in problem-solving tasks during anodal stimulation over the right hemisphere compared to sham or reverse (i.e., cathodal) stimulations (Chi and Snyder, 2011, 2012).

Most of the current scientific research for NIBS in cognitive sciences targets the DLPFC, considered for its pivotal role as a functional node among cortical and subcortical neuronal networks for decision-making processes, working memory, and cognitive flexibility. However, this trend of mainly focusing on this brain region may negatively impact on possible further information of the wide range of neuronal circuits involved in cognitive processes.

In general, NIBS applications in this field can be considered in an emerging and progressing point and more studies are required to corroborate the hypothesis that NIBS can be used to evaluate the underlying mechanisms of cognitive processes and to enhance their functions in both physiological and pathological aging. Indeed, NIBS may be considered as a reliable technique in this field not only for studying cognition but also for exploring the lateralization and locations of brain cognitive functions.

## Modulation mechanism of NIBS in Alzheimer's disease

Over recent years, several structural and functional techniques have been investigated to describe neural networks and their connections, considered as a broad brain map defined “connectome.” Nowadays it is well-known that NIBS techniques alter connectivity in both targeted and remote brain regions, inducing therefore local and distal effects. The integration of TMS with other techniques, such as electroencephalogram (EEG), fMRI, and positron emission tomography (PET), to study functional connectivity provides valuable details in specifically describing causal effects that neural assemblies mutually exert each other. The combination of TMS with EEG was introduced by Ilmoniemi et al. (1997); indeed, it has been postulated that the first EEG signal elicited by TMS (Transcranial Evoked Potentials = TEPs) reflects the excitability of the stimulated cortical region, namely its functional state, while the subsequent spatiotemporal distribution over the scalp of the stimulus-evoked wavelets depends on the propagation to other brain areas connected to the stimulated one. TMS-EEG recordings provide temporal resolution in the order of milliseconds (ms), from many scalp sites simultaneously and evaluate cortico–cortical interactions, tracking temporal dynamics and inner hierarchies of brain networks with two main advantages: (1) defining the causal interactions, either excitatory or inhibitory, between two brain regions; (2) examining the connectivity pattern of different cognitive activities related to specific tasks or brain states (Hallett et al., 2017; Rossini et al., 2020). However, a relevant disadvantage of this technique stems from the interaction between magnetic and stimulus-related brain responses, which can induce auditory-evoked potentials (AEPs) and sensory-evoked potentials (SEPs), and large-amplitude artifacts. Hence, specific control methodologies, data recording supervisions and artifact removal procedures are needed (Hallett et al., 2017). Notably, effective methods (e.g., neuronavigation systems) are now validated to minimize confounding factors and achieve reliable TMS-EEG measurements.

Another approach to investigate brain connectivity is through TMS combined with fMRI, which offers better spatial resolution compared to TMS-EEG, but with a poor temporal resolution due to the BOLD signal which is linked to the relaxation time of hemoglobin metabolites. Also tES in combination with fMRI can explore specific functional connectivity models and combine targeted stimulated regions with cognitive or motor processes (Rossini et al., 2019).

In animal models, a TUS protocol that outlasts the sonication period has been developed not to jeopardize tissue integrity, which is based on 30 ms bursts of ultrasound waves at 250 kHz, generated every 100 ms (10 Hz) for a 40 s time period producing effects on connectivity between pre-supplementary motor area and frontal polar cortex as measured by fMRI in the macaque (Verhagen et al., 2019). Similarly, brief bursts of ultrasound waves delivered at 10 Hz for 40 s targeted to the ventral posterolateral nucleus of the thalamus in pigs have yielded comparable outcomes (Dallapiazza et al., 2018). These findings underscore the potential for exploring the immediate consequences of the sonication period and for devising protocols aimed at modulating connectivity between brain regions that endure beyond the stimulation period.

In general, the combination of advanced procedures with NIBS techniques may provide original information on how NIBS interventions can induce neuroplasticity mechanisms and modulate cognition in elderly, which may accelerate the introduction of such methodologies into clinical practice (Klooster et al., 2024).

Until now, evidence in literature has supported the presence of cortical disconnection processes in people living with dementia, which also seems to begin at the earliest stages of the disease. Most studies have showed that dementia disorders including AD tend to correlate with functional connectivity alterations in the whole brain circuits rather than in a specific local region (Supekar et al., 2008; Vecchio et al., 2018, 2020; Rossini et al., 2019; Fathian et al., 2022). In this regard, together with other non-invasive and computational techniques, NIBS methods have widely showed their value in understanding neuronal dysfunctions in pathological brain networks providing remarkable scenarios for an early diagnosis and an increased knowledge of the pathological process in AD. Therefore, in the realm of neurodegenerative diseases (Table 2), pioneering TMS studies have explored cortical excitability, organization of motor maps and connectivity of primary motor areas (D'Amelio and Rossini, 2012; Menardi et al., 2022). A previous study demonstrated an increased motor cortex excitability and a frontal and medial shift of the excitable motor areas in AD patients (Ferreri et al., 2003). This evidence suggested the presence of neuronal reorganization in the cortical motor areas following pathological neuronal loss even in absence of evident motor deficits. Contrary or integrative to this interpretation, hyperexcitability may also be attributed to dysregulation of intracortical neurotransmitter concentration within inhibitory circuitries (i.e., GABA) or to compensatory mechanisms for silent synapses recruitment aiming to maintain functions (Francis et al., 1993; Ferreri et al., 2003). Additionally, central cholinergic circuits can be non-invasively tested using TMS by analyzing specific parameters such as short latency afferent inhibition (SAI), which has been shown to decrease in AD compared to non-cholinergic forms of dementia (Di Lazzaro et al., 2006). Although the mechanisms underlying these TMS findings remain partly unclear, these results pave the way for using TMS as a non-invasive tool to discriminate between different forms of dementia, improve *resilience mechanisms* to neurodegenerative challenges and evaluate disease progression in clinical and research settings.

**Table 2:** Non-invasive brain stimulation studies in Alzheimer's disease.

**References**	**Sample**	**NIBS technique**	**Target site**	**Main results**
**Transcranial Magnetic Stimulation**				
Brem et al. (2020)	34 AD	rTMS combined with cognitive training (sham-controlled study)	Motor cortex	The real group with cognitive training showed significant cognitive improvement compared to the sham group with sham cognitive training, but not compared to the real group with sham cognitive training
Cotelli et al. (2011)	10 AD	rTMS (sham-controlled study)	DLPFC	Compared to baseline or placebo, real treatment showed an improvement in language performances (also lasting effects 8 weeks after the end of treatment)
Cotelli et al. (2012)	Amnestic MCI	rTMS	Left parietal cortex	rTMS to the left parietal cortex improved memory performance in a MCI
Di Lazzaro et al. (2006)	20 AD, 20 FTD, and 20 HC	TMS	Motor cortex	SAI was normal in FTD patients, whereas it was reduced in AD
Di Lorenzo et al. (2016)	54 AD and 24 HC	TMS, cTBS, and iTBS	Motor cortex	AD pathology can be considered as a primarily disorder of LTP-like cortical plasticity not influenced by physiological aging and associated with a more impaired cognitive profile
Ferreri et al. (2003)	16 mild AD	TMS	Motor cortex	Motor cortex excitability was increased, and the center of gravity of motor cortical output showed a frontal and medial shift
Koch et al. (2012)	14 AD and 14 HC	cTBS and iTBS	Primary motor cortex	An impairment of LTP-like together with normal LTD-like cortical plasticity was observed in AD patients
Koch et al. (2022)	50 AD	rTMS (sham-controlled study)	Precuneus	Sessions of rTMS targeting precuneus may slow down cognitive and functional decline in Alzheimer's disease
Mencarelli et al. (2024)	16 AD	rTMS (sham-controlled study)	Precuneus	These preliminary results supported the possibility of using rTMS targeting the precuneus to arrest brain atrophy progression by manipulating network connectivity patterns
Motta et al. (2018)	60 AD and 30 HC	TMS and iTBS	Motor cortex	LTP-like cortical plasticity could be a reliable biomarker to assess synaptic impairment and predict cognitive decline in AD
Sabbagh et al. (2020)	131 AD	rTMS combined with cognitive training (sham-controlled study)	Broca's area; Wernicke's area; left and right dorsolateral prefrontal cortex; left and right inferior parietal lobule	Subjects with baseline ADAS-Cog ≤ 30 (mild AD) showed significant benefits after active rTMS and cognitive training
Traikapi et al. (2023)	4 AD	TMS	Bilaterally precuneus	An immediate treatment effect was observed in all patients' general cognitive functions assessed with ADAS, which was maintained and improved also at 3 months post-treatment
Vecchio et al. (2022)	72 AD	rTMS combined with cognitive training (sham-controlled study)	Broca's area, right and left DLPFC, Wernicke's area, right and left parietal somatosensory association areas	Significant improvement in cognitive scales. Delta and alpha1 SW seemed to be diagnostic biomarkers of AD, whereas alpha2 SW might represent a prognostic biomarker of cognitive recovery
**Transcranial Electrical Stimulation**				
Boggio et al. (2012)	15 AD	Bilaterally tDCS	Temporal regions	After patients received tDCS, their performance in a visual recognition memory test significantly improved
Dhaynaut et al. (2022)	4 AD	tACS	Bilateral temporal lobes	tACS seems to induce gamma activity in AD patients. A preliminary evidence of a possible effect brain protein clearance (specifically p-tau) was also observed after tACS sessions
Ferrucci et al. (2008)	10 AD	Anodal/cathodal tDCS (sham-controlled study)	Temporoparietal areas	After anodal tDCS, accuracy of the word recognition memory task increased, whereas after cathodal tDCS it decreased, and after sham tDCS it remained unchanged
Fileccia et al. (2019)	34 MCI	Anodal tDCS (sham-controlled study)	Left DLPFC	At follow-up, patients exposed to real anodal tDCS stimulation showed improvement in episodic verbal memory, in figure naming test, in a general index of cognitive function, and in a depression scale
Kim and Yang (2023)	16 AD	tDCS (sham-controlled study)	Frontal lobes	Significant improvement in cognitive domains in active treatment compared to the sham-tDCS group. A marked reduction in post-intervention plasma Aβ oligomerization tendency level was also observed in the active tDCS group
Pini et al. (2022)	22 AD and 23 bvFTD	Anodal or cathodal tDCS	Anodal target network: DMN in AD, SN in bvFTD; cathodal target network: SN in AD, DMN in bvFTD	In AD and bvFTD patients, cathodal tDCS showed behavioral improvement, whereas anodal tDCS led to cognitive improvement. Functional connectivity effects were not observed
Rodella et al. (2022)	33 individuals at early stage of cognitive impairment	Anodal tDCS combined with cognitive training	Left DLPFC	Improvement in working memory and attention/processing speed was observed after treatment and 6-months later in the group with real stimulation combined with cognitive training
Sprugnoli et al. (2021)	15 AD	tACS	Temporal lobes	tACS may increase brain oscillatory activity and blood perfusion in temporal lobes in AD patients
**Transcranial Ultrasound Stimulation**				
Beisteiner et al. (2020)	35 AD	TPS	DLPFC and areas of the memory (including default mode) and language networks	Improvement of neuropsychological scores after TPS treatment and it correlates with an upregulation of the memory network (fMRI data)
Dörl et al. (2022)	18 AD	TPS	Bilateral frontal cortex, bilateral lateral parietal cortex, and extended precuneus cortex	Visuo-constructive network nodes were not stimulated by TPS and their global efficiency was decreased, compatible with a natural progress of the disease. A correlation between visuo-constructive scores and changes in global efficiency was found
Jeong et al. (2021)	4 AD	tFUS	Right hippocampus	Mild improvement in measures of memory, executive, and global cognitive functions
Jeong et al. (2022)	8 AD	tFUS	Right hippocampus	No evidence of transient BBB opening was found after tFUS immediate recall and recognition memory were significantly improved on the verbal learning test and an increased glucose metabolism was observed in the right hippocampus
Matt et al. (2022)	18 AD	TPS	Bilateral frontal cortex, bilateral lateral parietal cortex, and extended precuneus cortex	Improvement in depression scale (BDI-II) after TPS therapy. A normalization of the functional connectivity between the SN and the ventromedial network was observed
Popescu et al. (2021)	17 AD	TPS	Bilateral frontal cortex, bilateral lateral parietal cortex, and extended precuneus cortex	Significant correlations were found between neuropsychological improvements and cortical thickness increase in AD-critical brain areas
Shinzato et al. (2024)	10 AD	TPS	Bilateral frontal cortex, bilateral lateral parietal cortex, and extended precuneus cortex	TPS significantly reduced neuropsychiatric symptoms after 30 days and after 90 days from treatment. A decreasing trend was also observed in global cognitive scores after 90 days, even if not statistically significant

AD, Alzheimer's disease; ADAS, Alzheimer's disease assessment scale; BBB, brain blood barrier; BDI-II, Beck depression inventory; bvFTD, behavioral variant of frontotemporal dementia; cTBS, continuous theta burst stimulation; DLPFC, dorsolateral prefrontal cortex; DMN, default mode network; fMRI, functional magnetic resonance imaging; FTD, frontotemporal dementia; HC, healthy controls; iTBS, intermittent theta burst stimulation; MCI, mild cognitive impairment; LTD, long-term depression; LTP, long-term potentiation; NIBS, non-invasive brain stimulation; tACS, alternating current stimulation; tDCS, transcranial direct current stimulation; tFUS, transcranial focused ultrasound stimulation; rTMS, repetitive transcranial magnetic stimulation; SN, salience network; SW, small world; TMS, transcranial magnetic stimulation; TPS, transcranial pulse stimulation.

Of note, a TBS protocol was applied in AD patients in comparison with HC individuals, showing an impairment of LTP-like together with normal LTD-like cortical plasticity in AD group (Koch et al., 2012). Similar findings were observed in the study by Di Lorenzo et al., where the Authors found that AD individuals with a more impaired LTP-like cortical plasticity presented a more severe cognitive decline. SAI was also impaired in AD, demonstrating a robust correlation with aging rather than with disease onset (Di Lorenzo et al., 2016). These results suggested that AD pathology may primarily present a LTP-like cortical plasticity dysfunction, which is not influenced by physiological aging and correlated with a major cognitive impairment.

In another study, LTP-like cortical plasticity and SAI were assessed by a TMS protocol to discriminate AD individuals from HC subjects. Only LTP-like cortical plasticity parameter resulted to identify AD pathology with high accuracy and it was also a significant indicator for disease evolution, supporting the hypothesis of using LTP-like cortical plasticity as a reliable biomarker for the disease and its progression (Motta et al., 2018).

Although tDCS-induced modulatory effects on functional connectivity have been widely explored and confirmed (Kunze et al., 2016), few studies have analyzed these effects in individuals with AD. Considering that memory improvement was observed in different studies targeting temporal regions with tDCS, it could be speculated that this cognitive improvement might be mediated by the restoration of functional connectivity (Ferrucci et al., 2008; Boggio et al., 2009, 2012). However, a recent study conducted by Pini et al. tried to test clinical and neurobiological effects of tDCS over default mode (DMN) and the salience (SN) networks in AD and behavioral variant of frontotemporal dementia (bvFTD) patients. Even though cognitive and behavioral improvements were observed, any significant effect was not detected for functional connectivity measurements (Pini et al., 2022). Taken into account these results, future studies should directly and specifically investigate the tDCS modulatory effects on neuronal functional connectivity in subjects with dementia.

Concerning other tES methodologies, in a study by Sprugnoli et al., the possibility to modulate cerebral perfusion by tACS applied on temporal lobes was assessed in mild to moderate AD subjects. An increased blood perfusion in these brain regions was observed after 1 h daily for 2 or 4 weeks sessions of tACS treatment (Sprugnoli et al., 2021). Additionally, 40 Hz (gamma) tACS sessions for 4 weeks (1 h for 5 days/week) targeting the bitemporal lobes showed an increased gamma spectral power on EEG recordings after treatment. Notably, an initial reduction of hyperphosphorylated tau (p-tau) clearance (about 2% of p-tau burden) was also observed in the mesial temporal regions of the majority of participants after tACS treatment (Dhaynaut et al., 2022).

As regards TPS, previous studies reported changes in functional brain connectivity, assessed by fMRI examinations, in the comparison between pre and post TPS treatment (Beisteiner et al., 2020; Popescu et al., 2021; Dörl et al., 2022; Matt et al., 2022). Beisteiner et al. (2020) showed in AD patients after TPS treatment an increased functional connectivity together with an enhancement of neuropsychological tests (i.e., CERAD scores) in specific memory networks such as hippocampus, parahippocampal cortex, precuneus, and parietal cortex. Similarly, TPS session seemed to alleviate depression symptoms in AD participants by decreasing functional connectivity between the ventromedial network and the SN (Matt et al., 2022). Interestingly, functional changes were observed in AD individuals by using graph analysis of a visuo-constructive network with fMRI. The study of Dörl et al. showed that non stimulated brain area, such as visuo-constructive networks, tended to reduce their functional connectivity accordingly with neuropsychological scores of visuo-constructive capacities (Dörl et al., 2022). These findings were in line with the natural progression of the disease and substantiated the efficacy of TPS in modulating cerebral networks and improving global cognitive status. However, further TUS studies on functional connectivity are needed to enhance the potential of such innovative techniques in the field of dementia.

Nowadays increased efforts are moving for searching for reliable biomarkers of both dementia onset and progression in order to find possible strategies (pharmacological and/or non-pharmacological) to prevent or slow down the disease course. Connectivity measurements assessed by NIBS methods seem to be emerging candidates to reach this goal. However, more studies and comparative analyses between different NIBS techniques may help in defining the best strategy for investigating brain network dysfunctions. In addition, further longitudinal and placebo-controlled studies are required to confirm previous findings and to validate NIBS methodologies in this field.

## NIBS in neurocognitive rehabilitation and Alzheimer's disease

Over the years, NIBS has emerged as a critical tool in the field of dementia research, particularly in addressing the complex and varied clinical profile of AD, which is characterized by a wide array of cognitive deficits and behavioral changes that progressively impact on daily global functioning. This diverse symptomatology reflects the involvement of multiple cognitive domains, including memory, attention, language, visuospatial skills, and executive functions (Buss et al., 2019; Teselink et al., 2021). The unique advantage of NIBS, both with TMS and tES, lies in its potential to modulate specific brain regions and networks that underlie these affected domains. Research suggests that targeted NIBS protocols can improve memory performance by modulating regions associated with episodic memory, such as the hippocampus and prefrontal cortex, or enhance executive functioning by stimulating the DLPFC (Menardi et al., 2022). Similarly, by addressing the neural circuits linked to affective and behavioral symptoms, NIBS holds promise for reducing agitation, apathy, and depressive symptoms, which are common symptoms in AD which significantly impact on the quality of life (Buss et al., 2019; Teselink et al., 2021).

NIBS can be applied for restoring or compensating cognitive functions by reorganizing functional networks and brain circuits via three main mechanisms: (1) strengthening of cortical excitability through a progressive readjustment induced by changes in synaptic plasticity; (2) activation of perilesional or contralateral regions to rebalance transcallosal brain networks and compensate the impaired function; (3) restoration of damaged neurons through the release of brain-derived neurotrophic factors which promote cell survival (Miniussi and Rossini, 2011).

As regards mild cognitive impairment (MCI) and AD (Table 2), several NIBS protocols showed their effects in improving memory and general cognitive functioning, although variable outcomes were observed among studies (Bystad et al., 2016, 2017; Chang et al., 2018; Petrovskaya et al., 2023).

A sham-controlled study explored the effects of 30 days rTMS treatment, showing that combining rTMS with cognitive exercises may enhance cognitive status in AD patients, whereas TMS-induced cortical plasticity at baseline may be a valid predictor for therapeutic outcomes. Indeed, the experimental arm receiving real rTMS and real cognitive training showed greater cognitive improvement compared to the sub-cohort undergoing sham rTMS and sham cognitive stimulation (Brem et al., 2020).

The potential of neuromodulation to improve memory impairments was assessed by other studies in AD patients and in MCI subjects (Boggio et al., 2009, 2012; Cotelli et al., 2012; Manenti et al., 2012; Wang et al., 2023). Considered the relevant role of PFC in cognitive functions, high-frequency sessions of rTMS on the DLPFC showed beneficial effects in modulating cognition in AD in different studies (Dong et al., 2018). Stimulation of the left parietal cortex increased accuracy in an association memory task, also with long-lasting effects (up to 24 weeks), in amnestic MCI. Similar results were observed for language performances by Cotelli et al. (2011). Other studies (Sabbagh et al., 2020; Vecchio et al., 2022) evaluated the efficacy and safety of a 6-week course of daily rTMS combined with cognitive training for treating mild to moderate AD patients. The therapy showed immediate cognitive improvements, especially in those with mild AD, and induced long-term brain connectivity changes, with EEG parameters serving as valuable biomarkers for diagnosis and recovery prediction. A further study explored the effectiveness of a novel treatment using 40 Hz TMS targeted to the precuneus bilaterally for 10 days to improve episodic memory and other cognitive functions. Results showed that three patients had an increased immediate word recall, whereas two showed improved attention skills: general cognitive function, as measured by the Alzheimer's Disease Assessment Scale (ADAS), improved immediately after treatment and after 3 months. This study suggested promising outcomes for using gamma-band TMS to enhance cognitive performances in AD patients (Traikapi et al., 2023).

In a randomized, double-blind, sham-controlled study by Koch et al., better cognitive performances were observed in AD participants after 24-week sessions of rTMS targeting precuneus (2 weeks of intensive treatment, daily for five times/week, followed by 22 weeks of maintenance phase, applied once weekly). In addition, in respect to those who performed sham stimulation, in the group treated precuneus cortical excitability remained unchanged and an increased local gamma activity was observed (Koch et al., 2022). A similar rTMS protocol, implemented with structural and functional MRI scans, showed a macro- and micro-structural maintenance and an increased functional connectivity in the precuneus in AD patients treated in comparison to the sham group (Mencarelli et al., 2024).

Regarding tES, in a randomized, double-blinded, sham-controlled study, tDCS method was investigated for its therapeutic potentials in AD subjects with amyloid PET positivity. Daily bi-frontal tDCS sessions were administered at home (2 mA, 30 min) for 12 weeks. Results showed that the active-tDCS group experienced significant improvements in cognitive functions related to language abilities, verbal memory, attention, and frontal functions compared to the sham-tDCS group. Additionally, there was a notable reduction in plasma Aβ oligomerization tendency in the active-tDCS group after treatment, suggesting therefore potential changes in AD-associated biomarkers (Kim and Yang, 2023).

In a previous study conducted on 10 AD patients, tDCS treatment delivered at temporo-parietal regions showed positive effects on performances in recognition memory in respect to sham treatment (Ferrucci et al., 2008). Similar results were obtained for visual recognition memory after anodal tDCS sessions (Boggio et al., 2012).

Another study by Fileccia et al. (2019) investigated the effects of tDCS targeting the left DLPFC in patients with MCI. Over 20 days, patients received daily sessions of 20-min anodal tDCS stimulation (2 mA). Compared to baseline and sham stimulation, the real tDCS group showed significant improvements in episodic verbal memory, figure naming, general cognitive functions, and depressive symptoms. Rodella et al. studied the effects of combining tDCS with computerized cognitive training over 12 sessions in 4 weeks targeting the left DLPFC. Results showed significant improvements in working memory, attention, and processing speed in the active tDCS group vs. the sham group. After 6 months, the group with real tDCS and cognitive training maintained working memory benefits, and their global cognitive scores, assessed by Mini-Mental State Examination (MMSE), did not decline (Rodella et al., 2022). Of note, these findings highlighted as combined interventions (i.e., real stimulation and cognitive exercises) may contribute to obtain more beneficial effects from treatment in preventing or delaying disease progression.

As regards novel TUS therapies, recent pilot studies reported 3-month long-term improvement in cognitive profiles and depressive symptoms in small group of mild to severe AD patients after sessions of TPS treatment (Beisteiner et al., 2020; Matt et al., 2022; Cheung et al., 2023). Promising outcomes were observed also in neuroimaging findings (Popescu et al., 2021; Dörl et al., 2022), including fMRI. Indeed, a decrease in cortical atrophy as well as an improvement in cognitive functioning was observed in AD subjects, suggesting that TPS may modulate cortical anatomy and the connected functions (Popescu et al., 2021).

A preliminary study investigated TPS as a potential non-invasive treatment for AD, with a focus on its impact on cognitive functions and behavioral symptoms. In this trial, 10 patients with mild to moderate AD were treated using TPS, and their progress was monitored over a period of 90 days. The results showed a significant reduction in neuropsychiatric symptoms, with notable improvements after 30 and 90 days from the stimulation period. Although there was a trend toward global cognitive improvements assessed by ADAS, this change was not statistically significant (Shinzato et al., 2024).

In general, the application of TPS has resulted to be well-tolerated by all participants with rare and transient side effects (i.e., headache or feelings of pressure at the stimulation site). Meanwhile, no major side effects or signs of neuronal tissue damage, assessed with structural MRI, have been observed. The ability to precisely modulate small brain areas excitability, open new and interesting avenues in modulating activity of nodes of cortical/subcortical networks sustaining cognition. Consequently, TPS has emerged as a promising add-on therapy for AD at various stages of the disease, boasting high safety and efficacy profiles.

Also tFUS is considered as an emerging method which seems to facilitate neuronal functions specifically related to the brain targets. Preclinical studies have suggested the therapeutic tFUS potential for AD patients by opening the blood–brain barrier (BBB), reducing amyloid pathology, and improving cognition. However, up to date, the efficacy and safety of this technique remain to be further established.

Apart from cognitive improvements, NIBS interventions seem to promote also non-neuronal changes, including alteration in BBB permeability and endothelial cells, mediated by different factors and signaling (e.g., VEGF, Ca^2+^ and NO levels), which are highly involved in AD pathology and can be considered as mediators for NIBS effects (Eguchi et al., 2018; Beisteiner et al., 2020; Petrovskaya et al., 2023). Preclinical investigations have suggested that AD drugs may exhibit enhanced efficacy following tFUS treatment, owing to transient increase of BBB permeability (Jordão et al., 2010; Aryal et al., 2015). A recent pilot study reported mild improvements in memory, executive, and global cognitive functions following tFUS targeting the right hippocampus in four patients with AD, without complaining adverse effects (Jeong et al., 2021). These data were confirmed in a larger AD cohort, where the effects of tFUS on BBB opening, regional cerebral metabolism, and cognitive function were explored. Interestingly, tFUS applied to the hippocampal structures demonstrated potential improvements in glucose metabolism and short-term memory, even in the absence of BBB opening (Jeong et al., 2022).

Hence, plasticity changes induced by neuromodulation may also be effective in the field of neurorehabilitation to improve cognitive performances in individuals with MCI and AD (Sandrini and Cohen, 2013). NIBS effects seem to be activity-dependent since they are notably enhanced and reinforced when combined with cognitive and behavioral activities to promote enduring outcomes. The aforementioned results supported the utility of these technique for treating cognitive and behavioral disturbance in individuals with cognitive decline and for using these tools as valid biomarkers to study the disease in terms of diagnosis and progression. However, further longitudinal and sham-controlled studies may enhance the accuracy and validity of all these outcomes in this field. In addition, a solid comparison between all these methodologies could help in finding the best interventional strategy to improve cognitive functioning in individuals with MCI and dementia.

## NIBS and future directions: artificial intelligence and brain-computer interface

In the last years, NIBS methods were combined with artificial intelligence (AI) for predicting with personalized parameters the disease onset as well as for investigating strategies tailored to individual patients to reach both effective treatments and accurate differential diagnoses (Khan et al., 2021; Carrarini et al., 2024). Computerized modeling approaches have been able to simulate the effects of NIBS on neural circuits and predict the spatial and temporal distribution of induced electric or magnetic fields in the brain. This provides deeper insights into how NIBS parameters may affect neuronal activities and networks. These models aim to standardize experiments and clinical trials, focusing on optimizing stimulation protocols and developing personalized stimulation strategies tailored to individual patients.

Moving toward these directions, sophisticated algorithms from large datasets, employing data-driven approaches such as deep-learning, have been introduced toward novel targets for stimulation and better efficacy of NIBS interventions across different patient populations (Xu et al., 2021; Li et al., 2022).

Recently, an Italian study assessed the effectiveness of TMS as a diagnostic tool for distinguishing between different neurodegenerative dementias. Participants underwent TMS assessments and the derived parameters [short-interval intracortical inhibition/facilitation (SICI-ICF), long-interval intracortical inhibition (LICI), and SAI] were analyzed using a random forest classifier, which achieved high accuracy, precision, recall, and F1 scores in differentiating the neurodegenerative disorders, indicating its potential as a useful diagnostic tool in clinical practice (Benussi et al., 2020).

Apart from differential diagnoses, novel AI techniques may be applied to investigate disease course and treatment responses. For instance, a study by Kayasandik aimed to optimize TMS treatment by predicting patient responses using machine-learning analysis of EEG signals (Kayasandik et al., 2022). Utilizing a support vector machine (SVM) classifier and employing feature selection methods, the study indicated a stronger correlation between TMS outcomes and post-treatment EEG patterns compared to pre-treatment, achieving accuracies of 93% and 79%, respectively. Notably, TMS effects on EEG, particularly in the theta band, seemed to be significant indicators of patient response to treatment. Overall, these findings pave the way for optimizing personalized treatment approaches for AD patients by using NIBS techniques combined with AI algorithms. Early identification of EEG biomarkers associated with TMS responses could potentially enhance the effectiveness and efficiency of TMS interventions in AD management.

Similar results in this field have been achieved in another study aimed to identify key brain regions involved in AD and their responsiveness to tDCS combined with cognitive intervention (Andrade et al., 2023). Employing a random forest classifier, the study identified specific EEG biomarkers that predicted response to tDCS treatment. Results highlighted four critical brain regions (FC1, F8, CP5, Oz, and F7 channels) as potential biomarkers for predicting cognitive response to tDCS and cognitive intervention in AD patients. These findings underscore the potential of EEG features in guiding personalized treatment strategies for AD. Additionally, to optimize tDCS, Luppi et al. used a virtual brain network model of AD, simulating 20 different tDCS setups (Luppi et al., 2024). They found that the most effective configuration involved right parietal anodal stimulation with a contralateral supraorbital cathode. A pilot study by Albizu et al. found that MRI-derived tDCS current models, analyzed using machine learning, could predict working memory improvements in older adults with 86% of accuracy (Albizu et al., 2020). Higher current intensity and specific directional patterns near the electrodes were associated with better cognitive outcomes, supporting the potential for personalized tDCS treatments.

Although promising results have been reached, these advanced AI models are currently more suited for scientific purposes than clinical applications.

Nowadays, numerous studies are also exploring the potential application of Brain-Computer Interface (BCI) technologies in rehabilitation and cognitive neuroscience. BCI devices, known for their accuracy, low cost, non-invasiveness, and affordability, have been integrated with TMS and other NIBS tools. BCI methods create one-direction communication devices, which collect and decode EEG signals in real-time sending this information to electronic systems or computers for environmental interactions (Folgieri, 2020). Indeed, controlling and modulating EEG brain activity to induce neuroplasticity is a key aim of BCI applications in NIBS methodologies.

Encouraging evidence from the field of neurorehabilitation shows the effective results of BCI combined with NIBS in promoting recovery in cognitive decline. Further data from clinical trials are needed to confirm the efficacy and safety profiles of this technology, to identify individuals who will benefit most from these interventions, and to investigate the long-term effects (Pichiorri and Mattia, 2020).

## Conclusions

Conceptually, due to their multidisciplinary and translational applications, NIBS approaches may represent a promising opportunity in cognitive sciences and physiological/pathological brain aging to utilize neuromodulation as an individualized intervention in patients with dementia, such as AD.

The integration of NIBS techniques with AI and computer science, including computational modeling, data analysis, and BCI, may offer interesting objectives and goals for both clinical practice and neuroscience research. By using such technologies, researchers and clinicians can gain deeper insights into the neurophysiological mechanisms of the brain and optimize the efficacy of NIBS for more personalized interventions in managing and treating brain disorders such as neurodegenerative diseases.
